# Altered cell cycle regulation helps stem-like carcinoma cells resist apoptosis

**DOI:** 10.1186/1741-7007-8-63

**Published:** 2010-05-25

**Authors:** James Chappell, Stephen Dalton

**Affiliations:** 1 Paul D Coverdell Center for Biomedical and Health Science, Department of Biochemistry and Molecular Biology, University of Georgia, 500 DW Brooks Drive, Athens, GA 30602, USA

## Abstract

Reemergence of carcinomas following chemotherapy and/or radiotherapy is not well understood, but a recent study in *BMC Cancer *suggests that resistance to apoptosis resulting from altered cell cycle regulation is crucial.

See research article: http://biomedcentral.com/1471-2407/10/166

## Tumor recurrence: a poorly understood phenomenon

Typically, when a tumor is discovered, patients are treated with a combination of surgery, chemotherapy and radiotherapy, depending on the location and type of cancer. The goal of these treatments is to completely remove or destroy any cancerous cell growth. Often these treatments are met with apparent success, as indicated by shrinkage in tumor volume. Tumor reemergence is, however, frequent [1] but this phenomenon is poorly understood. It is not even clear whether one common factor underpins the ability of many different tumor types to reemerge after a remission period.

This general problem has led researchers to investigate possible explanations for tumor recurrence. One idea is that tumor heterogeneity is derived from a cell capable of generating many different cell types. This has led to the prediction that at least some tumors arise from a cell with stem-like properties. It is unclear, however, whether this would be a naturally occurring multipotent stem cell or a cell that acquires stem-like properties following mutational events, for example. Although the origin of these stem-like cells is contentious, the term cancer stem cell (CSCs) is often applied and will be used here. CSCs are currently a 'hot' topic because their potential properties are consistent with a tumor-initiating cell that might be able to regenerate the complexity of an entire tumor [2]. The obvious question, however, is how CSCs could survive genotoxic damage under conditions in which other tumor cells die. The answer to this question could be crucial for the development of new tumor therapeutics.

## Subpopulations with increased apoptotic resistance

Understanding the mechanisms underpinning resistance to apoptosis following chemo- or radiotherapy has been prompted by the promise that this could lead to a new generation of therapeutic approaches. Several reports indicate that CSCs can pump out toxins through specific chemical efflux pathways [3]. These cells are often resistant to DNA damage and show greater DNA repair capacity than their non-stem-like counterparts [4]. New findings reported in *BMC Cancer *[5] reveal that altered cell cycle regulation could also have a role in apoptotic resistance in at least some CSCs.

Epithelial tumors are the most commonly diagnosed cancer type [6] and are therefore a high priority in terms of therapeutic development. Mackenzie and colleagues [5] address the problem by focusing on human head and neck squamous cell carcinomas (HNSCCs). HNSCCs are epithelial tumors with a high rate of tumor recurrence following initial treatment [7], making them a logical choice for investigation of apoptotic resistance mechanisms. As in previous studies, subpopulations of cells with stem-like properties were isolated by fluorescence-activated cell sorting (FACS), using the cell surface glycoprotein CD44 as a marker. Assays comparing *in vitro *'tumor sphere' formation and clonogenicity demonstrated that cells expressing high levels of CD44 (CD44^high^) with a highly compact, holoclone colony morphology have stem-like characteristics of CSCs.

Mackenzie and colleagues [5] show that CD44^high ^cells are less sensitive to several chemical apoptosis-inducing agents than are CD44^low ^cells. Although previous studies showed that ABC transporters are expressed at high levels in these cells, the ability to pump out foreign chemicals is not the entire story when it comes to apoptotic resistance [8]. The current study [5], for example, shows that treatment with chemotherapeutics and radiotherapy is less effective at promoting apoptosis in CD44^high ^cells, thereby arguing against a role for ABC transporters.

## Increased apoptotic resistance is associated with a G2/M phase cell cycle block

What, then, is the reason behind the observed apoptotic insensitivity in CD44^high ^CSCs? Insight into this question came from observations made by Mackenzie and colleagues [5], who showed that CD44^high ^subpopulations of epithelial, breast and prostate carcinoma cells have a consistently higher proportion of cells in the G2 phase. Using pulse chase experiments involving 5-iodo-2'-deoxyuridine (IdU) labeling of cells in S phase followed by FACS analysis, cell cycle progression was then tracked through G2 back into G1. The conclusion of this study is that CD44^high ^cells spent a consistently longer time in G2.

**Figure 1:**
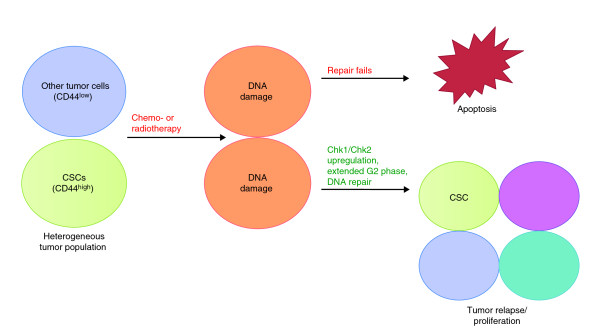
**Cancer stem cells (CSCs) escape apoptosis by upregulating G2/M checkpoint proteins, leading to a longer G2 phase and perhaps a longer window for DNA repair**. Following chemo- or radiotherapy, all cells undergo DNA damage, but the extended G2 phase in CSCs means that they have more time to repair the damage and are thus more resistant to apoptosis.

In normal cell types, DNA damage caused by chemicals, radiation or faulty replication halts cellular progression by activating a cell cycle checkpoint at the G2/M boundary, while DNA repair mechanisms restore genomic integrity. If the extent of damage is too great for repair mechanisms to correct, apoptosis is often induced [9]. Previous studies by Rich and colleagues [4] showed that glioblastoma cells are more resistant to radiotherapy than normal cells because they activate the DNA damage response. Checkpoint controls are implicated in this mechanism because inhibition of Chk1 and Chk2 checkpoint kinases remove the resistance to radiotherapy. A similar effect was observed by Mackenzie and colleagues [5], who found that levels of phosphorylated Chk1 and Chk2 are consistently higher in CD44^high ^subpopulations and thus greater checkpoint activation is achieved. Knockdown of Chk1 and Chk2 by chemical inhibition or by small interfering RNA targeting in CD44^high ^subpopulations led to a decrease in the percentage of cells in G2 phase to a level comparable to that of CD44^low ^cells. This provides further confirmation for a mechanism in which CD44^high ^CSCs, with a prolonged G2 phase, are apoptosis-resistant because of activated checkpoints. Activating checkpoint controls in CSCs therefore seems to impose a G2/M blockade, which gives a selective advantage in terms of cell survival and tumor-forming potential (Figure 1).

## Future directions and therapeutic value

Mackenzie and colleagues [5] have revealed important additional information about the mechanisms by which tumors evade current therapeutic treatments. Of interest is the finding that CD44^high ^subpopulations, displaying an increase in apoptotic resistance and a longer G2 phase, were also found in normal oral keratinocytes. This observation is a strong argument for the idea that a small subpopulation of cells with inherent stem-like properties naturally occurs and is not solely a product of malignant transformation.

More importantly, the authors [5] demonstrate that in addition to the efficient efflux and DNA repair mechanisms, CSCs also have an altered cell cycle program. Further biochemical characterization of these cells may explain how the G2/M checkpoint is activated and exactly how a longer G2 phase is conferring a survival advantage in cooperation with previously known mechanisms. A key question is what signaling pathways activate checkpoints in CD44^high ^cells. This could provide insight into what should be targeted for drug development. Prematurely releasing these cells from their prolonged G2 phase and G2/M block could trigger apoptosis instead of enhanced survival and tumor relapse. Therefore, it may be advantageous for future therapeutic treatments to target the Chk proteins or their upstream activators. In combination with modern chemical or radiation-based therapies, tumor elimination without relapse is an exciting possibility.
